# Novel Ti6Al4V Surface Treatment for Subperiosteal Dental Implants (Part II): Matrix Deposition and Osteogenic Markers

**DOI:** 10.3390/ma19081522

**Published:** 2026-04-10

**Authors:** Valentina Schiavoni, Lucia Memé, Giovanni Tossetta, Daniela Marzioni, Fabrizio Bambini, Andrea Frontini, Chiara Santoni, Paolo Moretti, Arianna Vignini, Roberto Campagna, Eleonora Salvolini

**Affiliations:** 1 Department of Clinical Sciences, Polytechnic University of Marche, 60121 Ancona, Italy; v.schiavoni@pm.univpm.it (V.S.); a.vignini@univpm.it (A.V.); e.salvolini@univpm.it (E.S.); 2 Department of Life Sciences, Health and Health Professions, Link Campus University Città di Castello (Pg), 06012 Città di Castello, Italy; l.meme@unilink.it; 3 Department for the Promotion of Human Science and Quality of Life, San Raffaele Roma University, Via di Val Cannuta, 247, 00166 Rome, Italy; giovanni.tossetta@uniroma5.it; 4 Department of Experimental and Clinical Medicine, Polytechnic University of Marche, 60126 Ancona, Italy; d.marzioni@univpm.it; 5 Department of Life and Environmental Sciences, Polytechnic University of Marche, 60121 Ancona, Italy; a.frontini@univpm.it (A.F.); c.santoni@pm.univpm.it (C.S.); paolo.moretti@staff.univpm.it (P.M.)

**Keywords:** Ti6Al4V, COL1A1, SPARC, DMP1

## Abstract

In a previous study, we demonstrated that a novel surface treatment applied to laser-melted Ti6Al4V substrates supports osteoblast-like cell adhesion, proliferation, and the activation of early osteogenic pathways. Building on these preliminary findings, the present work aimed to further investigate the ability of the same surface to promote extracellular matrix (ECM) deposition, organization, and osteogenic maturation, which are critical events for the establishment of a stable bone–implant interface in subperiosteal dental implants. Human osteoblast-like MG-63 cells were cultured on Ti6Al4V discs subjected to different surface treatments, including a proprietary surface modification (ATcs) specifically designed for subperiosteal applications. ECM formation and maturation were evaluated through scanning electron microscopy coupled with energy-dispersive spectroscopy, immunofluorescence, and semiquantitative analyses of osteogenic markers type I collagen (COL1A1), secreted protein acidic and rich in cysteine (SPARC), and dentin matrix protein 1 (DMP1) through Western blotting. The results showed that, while all tested surfaces supported cell adhesion, the ATcs surface promoted a distinct osteogenic profile characterized by enhanced DMP1 expression, organized collagen deposition, and the formation of calcium–phosphate–rich mineralized structures. Compared to surfaces that primarily stimulated cell proliferation or early matrix production, ATcs appeared to favour progression toward late-stage osteogenic maturation and matrix mineralization. Taken together, these findings extend our previous observations and indicate that this novel surface treatment not only supports osteoblast viability and early differentiation but also promotes extracellular matrix maturation, a key prerequisite for effective osseointegration. Although further in vivo studies are required, the present data provide additional biological rationale for the use of ATcs-treated Ti6Al4V surfaces in next-generation custom-made subperiosteal implant designs.

## 1. Introduction

Osseointegrated implant therapy represents a well-established and widely adopted solution for oral rehabilitation, with long-term clinical success depending largely on the quality and quantity of the supporting bone as well as on the stability of the bone–implant interface [1,2]. In clinical scenarios characterized by severe bone atrophy, conventional endosseous implants may not be feasible without extensive regenerative procedures, which are often complex, time-consuming, and associated with variable outcomes [3,4,5]. In this context, subperiosteal implants have re-emerged as a viable alternative, particularly with the advent of advanced imaging technologies, digital planning, and additive manufacturing techniques that allow the fabrication of patient-specific devices with improved biomechanical precision [6,7,8,9].

In line with this technological evolution, a fully digital workflow has been developed for the production of next-generation custom-made subperiosteal implants (EagleGrid^®^, Bergamo, Italy). This protocol is based on cone-beam computed tomography (CBCT) acquisition using standardized anatomical reference points, followed by virtual implant design through dedicated planning software. Such an approach allows precise assessment of bone anchorage sites and biomechanical load distribution prior to manufacturing, ultimately enabling the fabrication of individualized titanium grids that conform closely to patient-specific anatomy while minimizing internal stress concentrations [10,11].

Modern subperiosteal implants, typically manufactured from Ti6Al4V alloys using laser melting technologies, present unique biological challenges compared to endosseous implants [12]. Ti6Al4V implants have been reported to exhibit superior corrosion resistance, particularly under oxidative and acidic conditions simulating post-implantation inflammatory environments [13]. Rather than relying on deep intrabony anchorage, their long-term stability depends primarily on rapid and effective integration at the bone surface. Consequently, surface properties that can actively modulate osteoblast behaviour and promote osteogenic maturation are of critical importance. In recent years, considerable attention has been devoted to surface modifications aimed at enhancing cellular adhesion, proliferation, and early osteogenic differentiation on titanium-based biomaterials [14,15,16,17]. These approaches include micro- and nano-scale topographical modifications, chemical treatments, and biofunctional coatings designed to improve early cell–material interactions. However, most of these strategies have been primarily evaluated in terms of early cellular responses, while their ability to support later stages of osteogenesis remains less clearly defined. In particular, the processes of extracellular matrix deposition, organization, and mineralization, which are essential for the establishment of a stable bone–implant interface, have been comparatively less investigated.

In our previous study, we demonstrated that a novel surface treatment applied to laser-melted Ti6Al4V substrates supports osteoblast-like cell adhesion, proliferation, and the activation of early osteogenic pathways [18]. While these findings provided important preliminary evidence of biocompatibility and osteogenic potential, they did not address later stages of osteogenesis that are directly involved in the establishment of a stable and functional bone–implant interface. In particular, the ability of the surface to support extracellular matrix deposition, maturation, and mineralization remained to be elucidated. Therefore, the aim of the present study was to extend our previous observations by investigating the effects of the same Ti6Al4V surface treatments on extracellular matrix formation and osteogenic maturation. More specifically, we analyzed the expression and organization of key osteogenic and matrix-related markers, including type I collagen (COL1A1), secreted protein acidic and rich in cysteine (SPARC), and dentin matrix protein 1 (DMP1). Moreover, we evaluated the morphology of osteoblast-like cells adhering on discs subjected to different surface treatments and we assessed the composition of extracellular deposits. By focusing on these late-stage osteogenic events, this work seeks to provide a more comprehensive biological characterization of the novel surface treatment and to further clarify its potential relevance for next-generation custom-made subperiosteal implant applications.

## 2. Materials and Methods

### 2.1. Atomic Force Microscope (AFM)

AFM measurements were carried out on an AIST-NT Scanning Probe Microscope (Horiba Scientific, Kyoto, Japan). Images were acquired in non-contact mode with a pyramidal silicon tip of radius 8 nm. Images were performed in triplicate and analyzed with Gwydion software v 2.67 [19].

### 2.2. Experimental Design

Human osteoblast-like MG-63 cells were cultured in direct contact with titanium alloy substrates in order to assess the biological response to different surface modifications.

Cells were seeded onto circular discs fabricated from grade 5 Ti6Al4V alloy, each with a diameter of 10 mm. The titanium specimens were produced by New Ancorvis S.r.l. (Bargellino di Calderara di Reno, Italy) and subsequently subjected to distinct surface finishing procedures carried out by Al Ti Color S.r.l. (Piazzola sul Brenta, Italy). Five experimental groups were defined according to the surface finishing protocol applied to the discs. Untreated discs were used as the control group (CTRL). Discs polished by electroerosion were designated as EE. A third group consisted of discs subjected to a combined etching and sandblasting treatment (ES). In addition, discs treated with a proprietary surface modification newly developed by Al Ti Color, at which confidentially restrictions apply, were identified as ATcs. Finally, discs processed by color anodizing were classified as CA. After treatments, all specimens were washed with neutral surfactant, plasma decontaminated, packed in a clean chamber, and stored at room temperature before use. The experimental procedures were conducted using a blinded study design: investigators responsible for cell culture and biological analyses were not informed of the specific surface treatment associated with each disc until completion of the experimental phase.

### 2.3. Cell Culture

The human osteosarcoma-derived MG-63 cell line, commonly employed as an osteoblast-like in vitro model, was sourced from the American Type Culture Collection (ATCC; Manassas, VA, USA). Cells were maintained in a high-glucose formulation of Dulbecco’s Modified Eagle’s Medium (DMEM; Euroclone, Pero, Italy) supplemented with 10% (*v*/*v*) fetal bovine serum and penicillin–streptomycin 1X (Euroclone, Pero, Italy). Cell cultures were incubated under standard conditions at 37 °C in a humidified atmosphere containing 5% CO_2_ and routinely monitored to ensure optimal growth and viability.

### 2.4. Scanning Electron Microscopy (SEM) and Energy Dispersive X-Ray Spectroscopy (EDS)

3 × 10^4^ MG-63 cells were seeded onto titanium surfaces and incubated for 72 h. Culture medium was discarded and samples were fixed in 2.5% glutaraldehyde (Merck KGaA, Darmstadt, Germany) in 0.1 M sodium cacodylate buffer (Merck, Darmstadt, Germany) pH 7.4, post-fixed in 1% osmium tetroxide (Merck, Darmstadt, Germany) and completely dehydrated in increasing ethanol concentrations and hexamethyldisilane (Merck, Darmstadt, Germany). The specimens were then mounted on aluminum stubs, gold-sputtered by Emitech K550 sputter coater (Quorum Technologies, Laughton, UK) and observed with a Zeiss SUPRA 40 SEM (Carl Zeiss, Oberkochen, Germany) equipped with a Bruker Quantax 200 Z10 Energy Dispersive X-ray Spectrometer (EDS) (Bruker, Berlin, Germany) for the assessment of cell adhesion and morphology and for the determination of the elemental distribution.

### 2.5. Western Blot Analyses

Immunoblotting experiments were performed as previously described [20]. Briefly, cell pellets corresponding to 2.0 × 10^6^ cells were lysed in 100 µL of ice-cold extraction buffer consisting of phosphate-buffered saline (PBS, pH 7.4) supplemented with 1% Nonidet P-40, 0.5% sodium deoxycholate, 0.1% SDS, 1 mM sodium orthovanadate, 1 mM phenylmethylsulfonyl fluoride (PMSF), and 2 µg/mL aprotinin. Cell disruption was achieved by repeated passage through a 30-gauge syringe needle to ensure complete homogenization. Lysates were clarified by centrifugation at 16,000× *g* for 10 min at 4 °C, and the resulting supernatants were collected for protein analysis. Total protein concentration was determined using the Bradford assay. Equal amounts of protein (30 µg per sample) were separated by electrophoresis on 12.5% sodium dodecyl sulfate–polyacrylamide gels (SDS-PAGE) and subsequently transferred onto polyvinylidene fluoride (PVDF) membranes. Following protein transfer, membranes were blocked using EveryBlot Blocking Buffer (Bio-Rad Laboratories, Hercules, CA, USA) and then incubated overnight at 4 °C with the appropriate primary antibodies. These included mouse monoclonal antibodies directed against human DMP1, (1:200; Santa Cruz Biotechnology, Inc., Dallas, TX, USA) and SPARC (1:250; Santa Cruz Biotechnology, Inc., Dallas, TX, USA), or rabbit polyclonal antibodies against COL1A1 (1:500; Santa Cruz Biotechnology, Inc., Dallas, TX, USA) and β-actin (1:200; Merck, Milan, Italy), used as a reference. After washing, membranes were incubated for 1 h at room temperature with horseradish peroxidase (HRP)-conjugated secondary antibodies, specifically goat anti-mouse IgG (1:5000) or goat anti-rabbit IgG (1:150,000) (Sigma-Aldrich, St. Louis, MO, USA). Immunoreactive bands were visualized by chemiluminescence using the SuperSignal™ West Femto Maximum Sensitivity Substrate (Thermo Fisher Scientific) and detected with a ChemiDoc™ XRS+ imaging system (Bio-Rad Laboratories) and ImageJ software v. 1.46 (Rasband, W.S., ImageJ, U.S. National Institutes of Health, Bethesda, MD, USA, https://imagej.net/ij/, 1997–2018) was used to quantify related signal intensity. Protein extracts were obtained by pooling three independent experiments. Western blot analysis was performed on the pooled samples. Densitometric quantification was carried out in triplicate on the same blot.

### 2.6. Fluorescence Microscopy

DMP1 protein expression was assessed by fluorescence and confocal microscopy analysis. MG-63 cells were seeded onto the different titanium surfaces at a density of 3 × 10^4^ cells per disc and cultured for 72 h under standard conditions. At the end of the incubation period, the culture medium was removed and adherent cells were fixed using 4% formaldehyde for 15 min at room temperature. Immunofluorescence staining was performed to detect DMP1. After fixation, cells were incubated with a primary antibody specific for DMP1 (1:50; Santa Cruz Biotechnology, Inc., Dallas, TX, USA), followed by an appropriate fluorophore-conjugated secondary antibody AlexaFluor 488 (Thermo Fisher Scientific), according to the manufacturer’s instructions. This staining protocol allowed the evaluation of osteogenic marker expression in adherent cells. For visualization of the actin cytoskeleton, samples were stained with Alexa Fluor Plus 647–conjugated phalloidin (Thermo Fisher Scientific, USA). The fluorophore was prepared at a 1:400 dilution in 1X PBS, and 700 µL of the staining solution was applied to each disc. Samples were incubated for 1 h under gentle agitation, followed by three washes with 1X PBS to remove excess stain. Nuclear counterstaining was achieved using ProLong Glass Antifade Mountant with NucBlue (Invitrogen, Thermo Fisher Scientific). A volume of 50 µL of mounting medium was placed on each glass slide, and the titanium discs were positioned with the cell-seeded surface facing the mountant. After a 15 min incubation at room temperature to allow mounting, samples were analyzed using a NIKON A1R confocal microscope (Nikon Corporation, Tokyo, Japan) equipped with a 20× objective working also on long distance and able to acquire images on thick supports. Image acquisition and processing were carried out using NIS-Elements software (version 5.21.00; Nikon).

### 2.7. Statistical Analysis

Results were analyzed using GraphPad Prism software 8.4.2 (GraphPad Software Inc., San Diego, CA, USA). Differences between groups (each consisting in three samples) were determined by a One-Way ANOVA test.

## 3. Results

### 3.1. Atomic Force Microscope (AFM)

Root Mean Square (RMS) roughness and Mean roughness were calculated from AFM images using Gwyddion software by averaging the results of three replicates for each sample. No significant differences in roughness were observed among surface-modified discs, while CTRL disc displayed significantly higher average RMS. The results are shown in Table 1.

### 3.2. Scanning Electron Microscopy (SEM) and Energy Dispersive X-Ray Spectroscopy (EDS)

**Figure 1:**
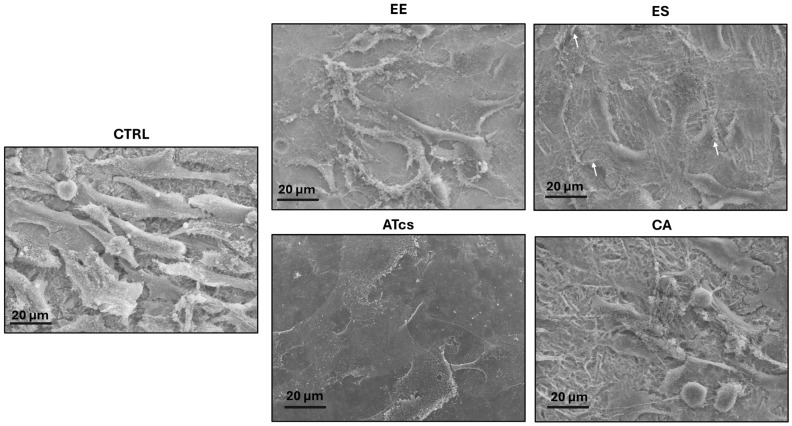
Representative SEM images of CTRL, EE, ES, ATcs, and CA discs colonized by MG-63 cells, showing the close contact and spreading of osteoblast-like cells on all the tested surfaces. Arrows indicate the presence of membrane ruffles.

**Figure 2:**
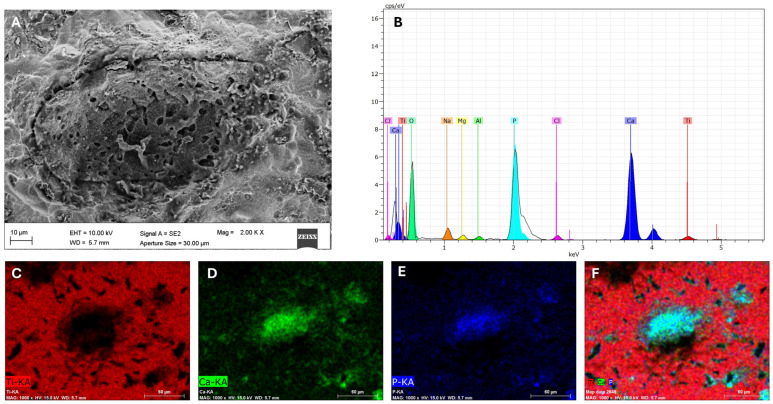
Scanning electron microscopy coupled with energy dispersive spectroscopy (SEM-EDS) analysis of ATcs disc following colonization by MG-63 cells. In the pericellular environment mineralized deposits (**A**) containing calcium and phosphorus, as shown by the characteristics EDS patterns (**B**), were observed. The compositional mapping evidenced that Ca (**D**) and P (**E**) were concentrated within the nodule and confirmed their co-localization (**F**), while showing the homogeneous distribution of Ti (**C**) across the dental material surface.

Scanning electron microscope analysis demonstrated that all the surfaces studied are biocompatible, since they allowed adhesion and proliferation of the osteoblast-like cells, in addition to preserving cell integrity and morphology. Concerning as-fabricated surfaces, their characterization by means of SEM was carried out in our previous study [18]. Figure 1 shows MG-63 cells closely in contact and spreading on top of all the tested specimens, although with some variability in cell shape and in cell–surface interactions across the five substrates (CTRL, EE, ES, ATcs, and CA) (Figure 1). It is noteworthy that these differences are not due to cytotoxicity, as confirmed by previously performed cell viability analysis [18]. In particular, on the CTRL surface MG-63 cells were partially aligned and exhibited a typical spindle-shaped morphology with the presence of short cellular protrusions indicative of their effective adhesion to the substrate. On the EE discs a lower cell density and a higher morphological heterogeneity were observed. Indeed, some cells appeared fusiform, while others exhibited a flattened polygonal shape, which suggest an enhanced adhesion to the dental material surface. On the sand-blasted acid-etched discs the SEM analysis evidenced an increased number of large cells forming an interconnected network and showing features suggestive of cell activation, such as membrane ruffles. Importantly, cells seeded on the ATcs surface formed a continuous and homogeneous layer of flat, polygonal cells in close contact with the substrate. The transition to a spread, flattened morphology is in fact intrinsically linked to adhesion strength, which is crucial for the osseointegration of the implant. Moreover, the morphological transformation from an elongated to a larger shape is indicative of cell differentiation into bone-forming cells. This is confirmed by the presence of mineral-like structures in the pericellular environment (Figure 2A). The CA surface displayed dense cellular networks but, next to polygonal and spindle-shaped cells, some rounded cells were observed, suggesting a poorer adhesion to the substrate.

In order to investigate the elemental composition of mineralized deposits formed on the ATcs disc surface, EDS analysis was performed (Figure 2). The SEM image revealed the presence of a well-defined, rounded structure with a porous surface in the extracellular matrix, suggestive of a mineralized nodule (A). The corresponding EDS spectrum (B) confirmed the presence of calcium (Ca) and phosphorus (P) as major elements, together with titanium (Ti) and other elements originating from the substrate, thus indicating the formation of a calcium–phosphate–rich deposit. These observations were further supported by the compositional mapping, which allows the visualization of the spatial distribution of the elements within the sample. Indeed, Ti was homogeneously distributed across the surface (C), while Ca (D) and P (E) signals were strongly enriched within the bone-like nodule. The merged compositional map (F) showed the clear co-localization of Ca and P within the nodule, consistent with a localized mineral deposition on the disc surface. The Ca/P ratio, calculated from the atomic percentages obtained by EDS analysis was 1.67, which is consistent with the atomic ratio range referring to hydroxyapatite [21]. The biological origin of these mineralized deposits is confirmed by their absence in cell-free discs, as shown in our previous study [18]. Overall, these results demonstrate the ability of the machined surface to support osteogenic activity and bone matrix mineralization.

### 3.3. Evaluation of Markers of Osteogenic Differentiation and Extracellular Matrix (ECM) Formation

**Figure 3:**
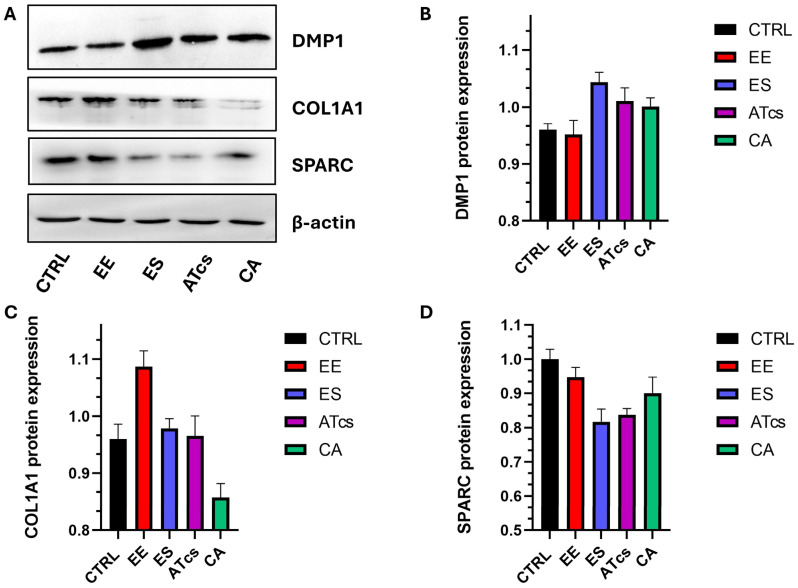
Representative Western blot analysis of osteogenic protein markers in CTRL, EE, ES, ATcs and CA groups. (**A**) Immunoblot bands showing the expression of DMP1, COL1A1 and SPARC, with β-actin used as the loading control. (**B**–**D**) Densitometric quantification of DMP1 (**B**), COL1A1 (**C**) and SPARC (**D**) protein levels normalized to β-actin. All values are expressed as the mean ± standard deviation of three technical replicates.

DMP1, COL1A1, and SPARC were selected as representative markers to evaluate osteogenic differentiation and extracellular matrix formation. Western blot analysis showed differential expression of osteogenic and extracellular matrix–related proteins in cells cultured on the different surfaces (Figure 3). DMP1 levels were increased in cells cultured on ES, ATcs, and CA discs compared to CTRL, suggesting enhanced osteogenic maturation under these conditions. COL1A1 was clearly expressed in CTRL and EE samples, while a progressive reduction was observed on ES and ATcs, consistent with a transition from early matrix deposition toward a more mature osteogenic phenotype, till a barely detectable band on CA. Notably, SPARC expression was enhanced on EE and CA, indicating active cell proliferation, while lower expression levels were detected in ATcs and ES. β-actin levels were comparable among all samples, confirming equal protein loading.

### 3.4. DMP1 Immunofluorescence and Confocal Analysis for Subcellular Localization

**Figure 4:**
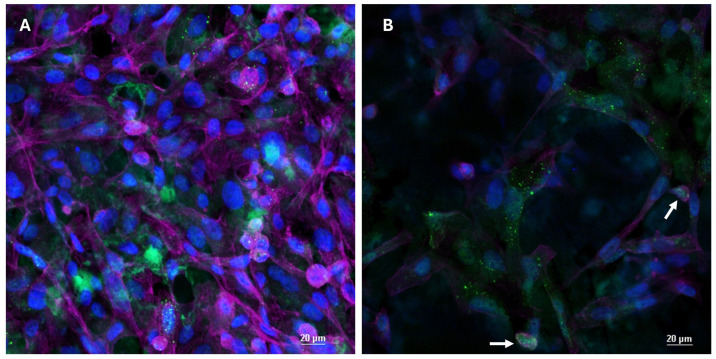
Representative images obtained by IF and confocal microscopy analysis performed on ES, ATcs and CA discs. DMP1 staining (green) localize mainly within the cytoplasm of many cells (the magenta colour was obtained by phalloidin used as cytoskeleton counterstaining). (**A**): z-stack was acquired to visualize the entire cells and to better describe DMP1 expression level. The staining pattern appeared to be spot-like but also diffused and mainly found within the cytoplasm of grouped cells visible on disc surface. (**B**): A single focal plan it is shown to better describe DMP1 subcellular localization, and some rare nuclear staining are visible (white arrows; nuclei are counterstained with Nuclear blue).

Immunofluorescence analysis revealed a clear DMP1 signal predominantly localized within the cytoplasm of cells cultured on the disc surface. DMP1 staining, counterstained with phalloidin to visualize the actin cytoskeleton, was mainly observed in grouped cells and displayed both a punctate (spot-like) and partially diffuse cytoplasmic distribution (Figure 4). Z-stack acquisition (Figure 4A) allowed visualization of the entire cell volume, confirming that DMP1 level expression was consistent in all positive cells. In the single focal plane image (Figure 4B), rare instances of nuclear DMP1 localization were detectable, as confirmed by nuclear counterstaining with Nuclear Blue. In contrast, DMP1 staining was very weak or nearly undetectable on the other supports CTRL and EE, in agreement with the low expression levels observed by Western blot analysis.

## 4. Discussion

The present study provides a detailed characterization of how distinct Ti6Al4V surface treatments modulate osteoblast-like cell behavior not only in terms of adhesion and proliferation, but more importantly in relation to ECM production, maturation, and mineralization. In a previous study, we reported preliminary evidence demonstrating that osteoblast-like cells are able to adhere and spread on the Ti6Al4V discs subjected to the different surface treatments. In the present work, while all investigated surfaces supported MG-63 cell attachment and spreading, as demonstrated by SEM analysis, the qualitative differences in cell morphology and matrix deposition indicate that surface chemistry and topography critically influence the progression of osteogenic differentiation. In particular, the ATcs surface appears to promote a biological response that is less proliferative and more oriented toward matrix maturation and mineral deposition, a combination that is highly desirable for stable bone–implant integration in subperiosteal applications. The SEM-EDS findings provide the first line of evidence supporting this interpretation. The identification of localized, calcium- and phosphorus-rich nodules on the ATcs surface strongly suggests the formation of mineralized extracellular matrix rather than nonspecific salt precipitation. These findings are consistent with previous ultrastructural studies showing that calcium–phosphate-rich nodules represent a characteristic feature of active matrix mineralization in osteoblast cultures [22,23,24,25]. The presence of these deposits indicates that the ATcs surface does not merely sustain osteoblast viability, but actively supports the biochemical conditions required for hydroxyapatite nucleation, a key event in functional osseointegration.

The analysis of COL1A1 expression further clarifies the differentiation stage of cells cultured on the different substrates. Type I collagen, encoded by COL1A1, constitutes approximately 90% of the organic bone matrix and is considered an early and mid-stage marker of osteoblast differentiation. Its synthesis is essential for providing the structural scaffold upon which mineralization subsequently occurs [26]. In the present study, COL1A1 expression was higher on CTRL and EE surfaces and progressively reduced on ES and ATcs surfaces. Rather than indicating impaired osteogenesis, this reduction is consistent with a transition beyond the early matrix-producing phase. Although reduced COL1A1 expression alone cannot be taken as definitive evidence of advanced osteogenic differentiation, in the present experimental context its decrease, together with increased DMP1 expression and the presence of calcium–phosphate-rich mineralized deposits, is consistent with progression from early matrix deposition toward a more mature mineralization-competent phenotype. Several studies have demonstrated that COL1A1 expression peaks during early osteoblast differentiation and decreases as cells progress toward matrix maturation and mineral deposition [27,28,29]. Therefore, the lower COL1A1 levels observed on ATcs surfaces suggest that osteoblast-like cells could have already completed the initial collagen deposition phase and have entered a more advanced stage of differentiation.

SPARC, also known as osteonectin, provides additional insight into the dynamic state of the extracellular matrix. SPARC is a matricellular protein involved in collagen fibrillogenesis, modulation of cell–matrix interactions, and regulation of growth factor signalling [30]. It is typically highly expressed in tissues undergoing active remodelling, where it contributes to matrix organization rather than serving as a structural component itself [31]. In bone, SPARC expression is associated with early osteoblast activity and proliferative phases of matrix turnover. SPARC protein levels peak during the early stages of differentiation and progressively decline as cells mature and begin to express markers of mature osteoblasts [32,33]. The elevated SPARC levels observed on EE and CA surfaces are consistent with a biologically active and remodelling-oriented environment, in line with previous reports linking SPARC expression to early osteoblast activity and matrix turnover [32,33]. Conversely, the reduced SPARC expression on ATcs surfaces suggests a relative decrease in matrix turnover and cellular proliferation, favoring stabilization of the deposited matrix. This shift is particularly relevant in implantology, as excessive or prolonged remodeling at the bone–implant interface may compromise long-term stability. It should be noted that the expression of COL1A1 and SPARC is not exclusively dependent on the stage of osteogenic differentiation. Surface-related factors, including cell–substrate interactions and mechanotransduction processes, may also contribute to the modulation of these proteins. In particular, surface roughness has been reported to influence osteoblast behavior and extracellular matrix production [34,35]. However, in the present study, AFM analysis did not reveal substantial differences in roughness among the surface-treated samples. Therefore, while a contribution of surface-related cues cannot be completely excluded, differences in roughness are unlikely to fully account for the observed expression patterns. In this context, the coordinated modulation of COL1A1, SPARC, and DMP1, together with the evidence of mineralized matrix deposition, supports the interpretation of a progression toward a more advanced osteogenic phenotype.

Among the analyzed markers, DMP1 emerges as the most informative indicator of osteogenic maturation in this study. Dentin matrix protein 1 is a non-collagenous phosphoprotein belonging to the SIBLING (Small Integrin-Binding Ligand N-linked Glycoprotein) family and plays a pivotal role in late osteoblast differentiation, osteocyte maturation, and regulation of mineralization [36]. DMP1 is critically involved in phosphate homeostasis and hydroxyapatite crystal formation, and its expression is tightly linked to the transition from osteoblasts to osteocytes embedded within mineralized matrix [37,38]. Genetic ablation of DMP1 results in severe defects in bone and dentin mineralization, underscoring its essential role in skeletal biology [39].

In the present work, DMP1 expression was markedly increased in cells cultured on ATcs surfaces, as demonstrated by both Western blot and immunofluorescence analyses. This finding is consistent with previous studies identifying DMP1 as a marker of late-stage osteogenic differentiation and mineralization [36,37,38]. This upregulation indicates that the ATcs surface actively promotes osteogenic maturation rather than merely enhancing early differentiation. The predominantly cytoplasmic and pericellular localization of DMP1 observed by confocal microscopy is consistent with its known role in matrix mineralization, where DMP1 is secreted and incorporated into the extracellular milieu to regulate crystal nucleation and growth [36]. The rare nuclear localization observed is also in agreement with emerging evidence suggesting that DMP1 may exert regulatory functions at multiple intracellular levels during osteocyte differentiation.

Importantly, the inverse relationship observed between COL1A1/SPARC and DMP1 expression across the different surfaces supports a temporal model of osteogenesis driven by surface properties. While CTRL and EE surfaces appear to favor early matrix production and remodeling, the ATcs surface appears to accelerate progression toward a mineralization-competent phenotype. This coordinated molecular pattern, together with the direct evidence of calcium–phosphate deposition, suggests that ATcs surfaces create a microenvironment favourable to functional osseointegration.

From a clinical and translational perspective, these findings are particularly significant for subperiosteal implant applications. Unlike endosseous implants, subperiosteal devices rely primarily on rapid and stable integration at the bone surface rather than on deep intrabony anchorage. While the ES surface induced the highest absolute levels of DMP1 expression, the ATcs surface promoted a more balanced osteogenic profile, characterized by coordinated modulation of extracellular matrix markers and clear evidence of mineralized matrix deposition. This suggests that, on ATcs surfaces, DMP1 upregulation is functionally coupled to extracellular matrix maturation and mineralization rather than representing an isolated molecular response. A surface capable of supporting such a controlled and homogeneous progression toward late-stage osteogenesis is therefore indicative of a biological environment favorable to bone–implant interface formation, which is known to be a key determinant of implant stability. However, whether these effects translate into improved mechanical stability and long-term clinical outcomes requires further in vivo and biomechanical investigation. In this context, the induction of DMP1 on ATcs surfaces represents a biologically meaningful indicator of improved bone–implant interface quality, supporting the rationale for the use of this novel surface treatment in next-generation subperiosteal implant designs. Nonetheless, while these findings highlight the promising osteogenic potential of the ATcs surface, further studies, including long-term and in vivo investigations, are required to fully elucidate the mechanisms underlying surface-driven osteogenic maturation and to confirm their relevance for clinical osseointegration.

## 5. Conclusions

The present study demonstrates that distinct Ti6Al4V surface treatments differentially modulate osteoblast-like cell behavior, with the ATcs surface promoting a coordinated shift toward extracellular matrix maturation and mineralization. This effect is supported by the combined evidence of increased DMP1 expression, reduced COL1A1 and SPARC levels, and the presence of calcium–phosphate-rich mineralized deposits. Together, these findings indicate that ATcs surfaces favor progression toward a mineralization-competent osteogenic phenotype rather than merely enhancing early differentiation. From a translational perspective, such a biological profile is particularly advantageous for subperiosteal implant applications, where rapid and stable surface integration is critical. Although further in vivo and long-term studies are required, the present data provide a strong biological rationale for the use of this novel surface treatment to improve bone–implant interface quality and support successful osseointegration.

## Figures and Tables

**Table 1:** Roughness values calculated from AFM images. Results are expressed as mean ± standard deviation of values obtained in three different areas of each disc.

Sample	Average RMS (nm)	SD	Average Mean Roughness (nm)	SD
**CTRL**	93.9	19.7	71.6	15.6
**EE**	57.3	15.7	43.8	11.5
**ES**	31.8	3.1	23.4	3.2
**ATcs**	59.5	15.5	45.4	10.8
**CA**	61.8	9.2	44.6	4.4

## Data Availability

The original contributions presented in this study are included in the article. Further inquiries can be directed to the corresponding authors.

## Abbreviations

The following abbreviations are used in this manuscript:

AFM	Atomic Force Microscope
ATcs	Al Ti Color surface
CA	Colour Anodizing
CBCT	Cone-beam computed tomography
COL1A1	Collagen Type I Alpha 1 Chain
CTRL	Control
DMP1	Dentin Matrix Protein 1
ECM	Extracellular Matrix
EE	Polished via Electroerosion
ES	Etching and Sandblasting
PMSF	Phenylmethylsulfonyl fluoride
PVDF	Polyvinylidene fluoride
RMS	Root Mean Square
SD	Standard Deviation
SDS-PAGE	Sodium dodecyl sulfate–polyacrylamide gels
SEM-EDS	Scanning Electron Microscopy coupled with Energy-Dispersive Spectroscopy
SPARC	Secreted Protein Acidic and Rich in Cysteine
